# Report of Mortality in Buffalo for the Month of June, 1861

**Published:** 1861-08

**Authors:** J. Whitaker

**Affiliations:** Health Physician


ART. V.—*Report of Mortality in Buffalo for the month of June,* 1861.
By J. WHITAKER, M. D., Health Physician.

The following tables show the number of deaths in the City of Buffalo for the month of June, 1861:

CAUSES OF DEATH.

| | | | |
|---|---|---|---|
| Abscess, | 4 | Diphtheria, | 2 |
| Accident, | 4 | Dropsy, | 4 |
| " drowning, | 4 | Fever, | 1 |
| Albuminuria, | 1 | Fever Puerperal, | 3 |
| Anæmia, | 2 | " Remittent, | 2 |
| Aneurism, | 1 | " Scarlet, | 8 |
| Angina, | 2 | " Typhoid, | 3 |
| Apoplexy, | 1 | Hemorrage from Lungs, | 1 |
| Brain, compression of | 1 | Inflammation of Bowels, | 1 |
| Cancer of Stomach, | 1 | " of Meninges, | 4 |
| Cholera Infantum, | 1 | " of Larnyx, | 2 |
| Consumption, | 6 | " of Lungs, | 2 |
| Convulsions, | 8 | Inf. of Peritoneum, | 2 |
| Croup, | 5 | Marasmus, | 3 |
| Debility, | 2 | Old Age, | 4 |
| Delirium Tremens, | 3 | Premature Birth, | 1 |
| Diarrhœa, | 2 | Rheumatism, | 1 |
| Disease of the Brain, | 2 | Small Pox, | 3 |
| " " Heart, | 1 | Still Born, | 8 |
| " " Lungs, | 4 | Whooping Cough, | 3 |
| " " Spine, | 2 | Unknown, | 8 |
| Total, | | | 123 |

AGES.

| | | | |
|---|---|---|---|
| 1 year and under, | 33 | Between 40 and 50 years, | 11 |
| Between 1 and 3 years, | 13 | Between 50 and 60 years, | 4 |
| Between 3 and 5 years, | 7 | Between 60 and 70 years, | 3 |
| Between 5 and 10 years, | 15 | Between 70 and 80 years, | 2 |
| Between 10 and 20 years, | 7 | Over 80, | 3 |
| Between 20 and 30 years, | 13 | Unknown, | 3 |
| Between 30 and 40 years, | 9 | | |
| Total, | | | 123 |

SEX.

| | | | |
|---|---|---|---|
| Males, | 67 | Females, | 56 |

COLOR.

| | | | |
|---|---|---|---|
| White, | 121 | Colored, | 2 |

NATIVITIES.

| | | | |
|---|---|---|---|
| Born in the United States, | 80 | Born in England, | 3 |
| " Germany, | 23 | " Scotland, | 1 |
| " Ireland, | 9 | " Canada, | 2 |
| " France, | 2 | " Unknown, | 3 |
| Total, | | | 123 |

REGISTER OF MORTALITY—Continued.

BY WHOM CERTIFIED.

| | | | |
|---|---|---|---|
| By Practitioners, | 94 | By Undertakers, | 21 |
| By Coroner, | 8 | | |
| Total, | | | 123 |

LOCALITY.

| | | | |
|---|---|---|---|
| City at large, | 104 | Foundling Asylum, | 7 |
| Sisters' Charity Hospital, | 2 | Erie Co. Almshouse, | 2 |
| General Hospital, | 5 | Small Pox, | 3 |
| Total, | | | 123 |

The average number of deaths during the month of June, for the last five years, is 124, showing a trifle above the mortality of the preceding month.

The cases of small pox which have occurred this year have all been imported and sporadic cases, and have been transported immediately to the Small Pox Hospital, which is the only locality in the City where the disease exists.

J. WHITAKER, Health Physician.

